# Case report: Movement analysis in oncological rehabilitation: proposal of a kinematic and surface electromyographic protocol in breast oncology

**DOI:** 10.3389/fnhum.2023.1272027

**Published:** 2024-01-23

**Authors:** Giulia Bongiorno, Andrea Tomasi, Giulio Vigni, Alessandro Rizzardo, Helena Biancuzzi, Francesca Dal Mas, Rym Bednarova, Luca Miceli

**Affiliations:** ^1^Friuli Riabilitazione Rehabilitation Center, Roveredo in Piano (PN), Italy; ^2^Papa Giovanni XXIII Hospital, Monastier (TV), Italy; ^3^Department of Economics, Ca Foscari University of Venice, Venice, Italy; ^4^Department of Management, Ca Foscari University of Venice, Venice, Italy; ^5^Collegium Medicum University of Social Sciences, Łódź, Poland; ^6^Pain Medicine, Hospital of Latisana (UD), Latisana, Italy; ^7^Centro di Riferimento Oncologico di Aviano (CRO) IRCCS, Aviano (PN), Italy

**Keywords:** kinematics analysis, breast cancer, personalized rehabilitation, surface electromyography, pulsed radiofrequency

## Abstract

**Introduction:**

Breast cancer disease often affects the ipsilateral shoulder joint, with pain and joint limitation. Proper pain management, which can be obtained using, for example, pulsed radiofrequency of the suprascapular nerve, can help the physiotherapist mitigate patient pain. The modern technologies of kinematic analysis and surface electromyography of movement analysis can give further support in building a personalized rehabilitation program, based on the quantitative study of movement, in this case of the upper limb.

**Methods:**

A brief case report was conceived to develop and test the evolution of a shoulder joint analysis protocol based on an inertial accelerometer and non-invasive surface electromyography.

**Results:**

An analysis algorithm was defined to adapt to the needs of patients operated on at the breast based on a kinematic component (ROM - range of movement - and Jerk index) and an electromyographic one (study of muscle behavior in groups of four). The coactivations were also evaluated, both as an average value and in graphical form, to offer the physiotherapist a complete overview of the movement of the upper limb.

**Discussion:**

The promising protocol results underline its strengths, including the simplicity of use, combined with the reduced time required for processing the reports and the portability of the PC-sensors complex, making these analyses potentially valuable for patient care.

## Introduction

Breast neoplastic pathology can lead to an alteration of the kinematics of the shoulder, mainly on the operated side, with painful symptoms and even persistent range of motion limitations, after surgery, often due to the surgical insult on the pectoralis major muscle (Shamley et al., 2007; Hage et al., 2014; Neto et al., 2018; Yang and Kwon, 2018). Shoulder kinematics after breast surgery may also worsen pain (Prieto-Gómez et al., 2020). Physiotherapy treatment aims to restore normal movement as much as possible, preferably working in harmony with pain medicine to allow patients to be subjected to manual therapies and then to perform the therapeutic exercises set up in the hospital at home. This is especially true for painful shoulder pathologies (Nakandala et al., 2021). Selecting the most suitable treatments for each patient is the aim of personalized rehabilitation. In this regard, non-invasive movement analysis techniques that exploit surface electromyography and inertial sensors can help to create reports available to physiotherapists (Cools et al., 2020). From this point of view, however, it is important to have intra-patient comparisons, for example before and after a specific treatment or procedure, and inter-patient comparisons between different subjects. To do this, the parameters investigated, especially the electromyographic ones, must be indexed on the MVIC (“maximum voluntary isometric contraction”) of the patients (Edwards et al., 2017). Regarding pain management, as an aid to rehabilitation, pulsed radiofrequency offers a minimally invasive and potentially long-lasting (weeks-months) approach with a single treatment (Luleci et al., 2011). Finally, fibromyalgia is a chronic and disabling pathology, which is complicated to treat and mainly affects women not of an advanced age. It leads to polyarticular pain, mainly in the shoulder girdle and pelvic level, almost always in the absence of radiological objectivity (Siracusa et al., 2021).

The literature states that breast cancer pathology has thus far received little attention from a movement perspective; the most extensive experience, which analyzed the electromyographic patterns of the shoulder surface in women undergoing breast surgery with and without pain and in healthy subjects, was published in 2020 (Prieto-Gómez et al., 2020). However, the analysis protocol used, in our opinion, had some critical issues. Firstly, a non-certified medical use synchronized camera (Microsoft KinectÓ) was used to capture the “start” phases of each movement cycle, forbidding the protocol’s applicability in clinical practice, which is outside the scope of research. Additionally, electromyographic analysis was not integrated with data derived from a synchronized multi-axial inertial sensor (IMU), able to measure joint movement angles and the fluidity of the movement itself. For these reasons, a specific protocol was developed, and the “start” phases of movements were extracted from the accelerometer signal. Furthermore, the described methodology does not allow for a high degree of automation in reporting test results, requiring a greater amount of time for the processing of data acquired from video analyses. The purpose of the study was to assess the ability of the proposed analysis protocol to detect kinematic differences, potentially useful for rehabilitation purposes, before and after analgesic treatment in breast surgery patients and between diseased and healthy subjects. Pulsed radiofrequency treatment on the suprascapular nerve was chosen as the intervention because it has a strictly local-regional effect and, therefore, the subsequent analysis is not subject to confounding factors (bias) arising from the sometimes unpredictable effects of pharmacological therapies (e.g., psychomotor slowing from opioids, drowsiness, and dizziness from serotonin reuptake inhibitors) used for pain relief in this patient category.

This brief report aims to describe the interaction of doctors and physiotherapists in proposing a kinematic and electromyographic surface analysis protocol potentially usable in patients with breast cancer to build a personalized rehabilitation program, working in the context of the “Oncology in Motion” project which characterizes the national Cancer center of Aviano (Italy) (Bednarova et al., 2022).

## Methods

The main subject-object of the kinematic and electromyographic analysis was a 45-year-old woman, affected by previous G3 phase infiltrating neoplasia in the left breast since 2019. She underwent neoadjuvant chemotherapy treatment with paclitaxel for 12 cycles. She then underwent a left quadrantectomy and lymphadenectomy in 2020. Subsequently, she underwent left thoracic radiotherapy. After 3 months, following the appearance of a suspicious pericicatricial nodule with the presence of atypical cells, the patient underwent a radical mastectomy followed by carboplatin-based chemotherapy treatment. The only significant anamnestic diagnosis of fibromyalgia was with migrating bilateral polyarticular involvement (shoulders and coxofemoral joints bilaterally) in the absence of radiological lesions on the shoulders. Ultrasound and Rx reported in the norm. Home therapy with duloxetine and opiates was recommended and then suspended due to ineffectiveness on painful fibromyalgia symptoms and due to the simultaneous onset of side effects (confusion, nausea, and dizziness). Currently, the patient is undergoing only sporadic use of NSAIDs (ibuprofen 600 mg bis in die) and acetaminophen 1 *g* orally twice a day as home therapy. No further anamnestic findings were reported by the patient, no other surgeries in the past, and no allergies were reported. The patient visited the pain medicine clinic due to severe, continuous pain, quantified in 8/10 on the NRS scale, both at rest and on movement, located in the right and left shoulders, with associated bilateral functional ankylosis.

**FIGURE 1:**
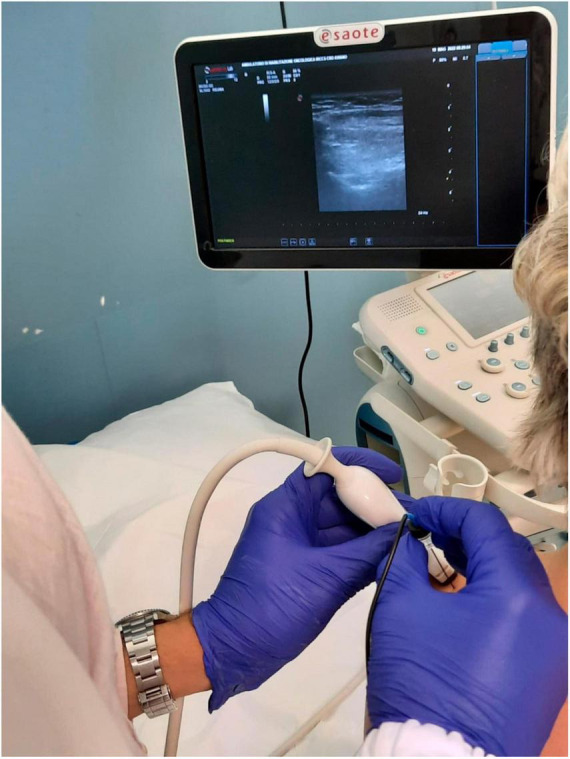
Performing pulsed radiofrequency treatment of the left suprascapular nerve (photograph obtained with the consent of the portrayed subject).

The SPADI (Breckenridge and McAuley, 2011) (shoulder pain and disability) index was 70/100 on the right side and 80/100 on the left side. The right and left ranges of motion (ROM) in abduction, flexion, and rotation, respectively, were 167° and 100°, 158° and 130°, and 122° and 106°. The patient had not undergone physiotherapy in the last 6 months due to the pain and reported that, in this period, the pain in the right shoulder had also worsened, although she had not been involved in the oncological treatment path. After obtaining written informed consent, the patient underwent two treatments with pulsed radiofrequency (TherMedico NK 1 radiofrequency lesion generator^®^ Germany) in a day hospital Figure 1. The procedure was performed first on the left suprascapular nerve and also on the right side after 2 weeks, as an analgesic treatment for the fibromyalgia pathology that had not been responsive to medical therapy. A 100 mm long needle was used, under sterile conditions, with continuous ultrasound guidance. The treatment, at 35 V with the needle tip temperature always lower than 42°C, lasted 5 min for each side. Impedance values were 1100 Ohm, sensory nerve stimulation was 0.6 V, and the motor was at 1 V during needle placement. The choice of pulsed radiofrequency treatment was due to the ineffectiveness of pharmacological therapy on shoulder pain in the past and the need to bring the patient back to an adequate physiotherapy course as soon as possible. For this reason, it was decided to resort to peripheral nerve neuromodulation, which has the advantage of reducing the perception of pain without reducing the motor function of the treated nerves (Erdine et al., 2009), a particularly important fact in the rehabilitation field. The patient was also subjected, after obtaining informed consent, to a kinematic analysis (measurement of the range of joint movement and evaluation of the jerk index bilaterally) and surface electromyography on both shoulders before and after the pulsed radiofrequency procedures. The measurements were collected the day before the first neuromodulatory treatment and 4 weeks after the second, when pulsed radiofrequency generally reaches its analgesic efficacy (Dey, 2021). The instrumentation used was a triaxial Bluetooth accelerometer (200 Hz G-sensor bioengineering^®^ Garbagnate, Italy) fixed at the mid-humeral level using an elastic band and a surface electromyograph at 1000 Hz frequency (Freemg 1000 BTS bioengineering^®^ Garbagnate, Italy) positioned on the muscles to be investigated.

**FIGURE 2:**
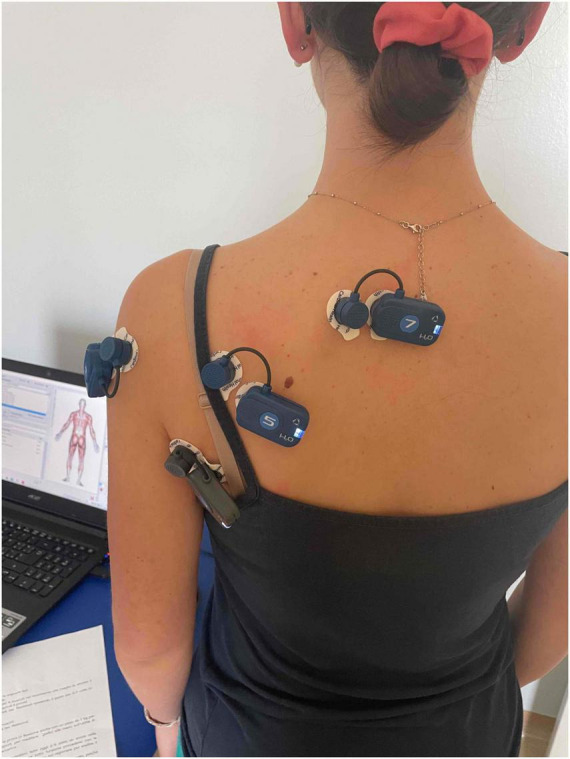
Example of positioning of the electromyographic probes (photograph obtained with the consent of the portrayed subject).

The electrodes (Kendall Arbo^®^, 24 mm in diameter) were applied to the muscles investigated using the SENIAM guidelines (Hermens et al., 2000; Figure 2).

**FIGURE 3:**
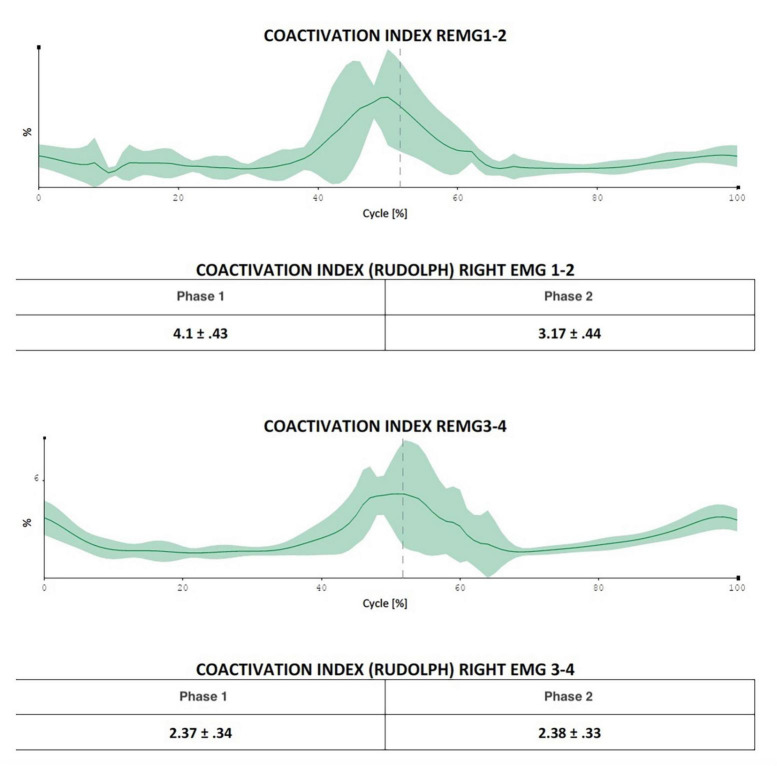
The figure indicates the possibility of evaluating in which exact moment of the movement cycle the coactivations manifest themselves most (in the figure in the central phase of the movement, a fact that is difficult to extrapolate using only Rudolph’s formulas).

In the electrode application region, the skin was washed, gently rubbed with sandpaper, and cleaned with alcohol. The pairs of electrodes for each electromyographic probe were positioned maintaining about 20 mm between them, longitudinally aligned concerning the muscle fibers, around the midpoint of the muscle belly. Movements analyzed were forward flexion of the upper limb with the forearm extended, starting from the vertical position with the patient standing and the arm close to the trunk; abduction of the arm with the forearm extended starting from the vertical position with the patient standing and the arm close to the trunk; and humeral rotation starting from the position with the arm abducted at 90° and the forearm flexed on the humerus at 90°, always with the patient standing. Muscles analyzed for flexion and abduction were anterior and middle deltoid, upper, middle, and lower trapezius, pectoralis major, teres major, and infraspinatus. Two separate sessions were carried out to analyze the muscles. Four sEMG probes were used to evaluate the percentage contribution of the muscles for each acquisition (flexion and abduction). The acquisitions were carried out on the same day: anterior and middle deltoid and upper and lower trapezius (first group); pectoralis major, middle trapezius, infraspinatus, and teres major (second group). For rotations, we chose to investigate the teres major, infraspinatus, posterior deltoid, middle trapezius (first group), latissimus dorsi, pectoralis major, teres major again, and clavicular head of the pectoralis major (second group). The software used for the analysis was EMG-analyzer (bioengineering^®^ Garbagnate, Italy), installed on a personal computer equipped with a Windows^®^ 10 professional operating system. The data acquisition protocol first envisaged an acquisition of the electromyographic signal during three-second maximal isometric contraction (MVIC, maximum voluntary isometric contraction) for each muscle investigated, according to SENIAM guidelines (Hermens et al., 2000). Subsequently, the patient performed a minimum of five cycles for each movement investigated for the right and left side for each of the three foreseen movements. Flexion, abduction, and rotation of the arm, in a standing position, were studied. Each cycle involved bringing the limb to the starting position described above for each of the three movements, pausing for 3 s. Forward flexion, abduction, and rotation were performed as linearly as possible and at a constant speed to the maximum achievable degree. The patient was then asked to return to the starting position, pausing for about 3 sec, and then restarting. The two phases of outward and return that characterize the cycle correspond, respectively, to the phase of reaching the maximum joint angle (with a momentary acceleration stop) and to the movement back to the starting position of the upper limb. The report returned, for these cyclicities, an average of the range of motion (ROM), an index of fluidity of the gesture [Jerk index (Beirens et al., 2021)], the electromyographic activity for each of the muscles investigated during the cyclicity of the movement, both as an absolute value and indexed on MVIC%, and the coactivations of the muscles investigated in pairs, both as an average value [Rudolph index (Rudolph et al., 2000)] in the outward and return phases and in graphical form over time (Ranavolo et al., 2015; Figure 3).

The muscle pairs investigated for coactivations were, for abduction and anterior flexion, anterior deltoid-middle deltoid, upper trapezius-lower trapezius, pectoralis major-middle trapezius, and infraspinatus-teres major. For rotation, the muscle pairs investigated were teres major-infraspinatus, posterior deltoid-middle trapezius, pectoralis major-teres major, and the clavicular head of pectoralis major-latissimus dorsi.

The role of muscle coactivation is to improve neuromuscular coordination and to increase the precision and stability of the movement. By calculating and knowing the electromyographic value of the first muscle contraction divided by the value of the second muscle contraction, the muscle activation index is determined. The same analyses were then performed on a 22-year-old healthy subject, after obtaining informed consent, in order to compare the data obtained from the patient.

## Results

After treatment with pulsed radiofrequency, the patient reported a significant improvement in pain symptoms - NRS of about 3/10 at rest and 4/10 with left movement, 4/10 at rest, and 5/10 with right movement. The improvement in percentage terms was 62% on the left at rest and 50% on movement, while on the right it was 50% at rest and 37% on movement, with a significant improvement in the SPADI index, reduced on the left from 80/100 to 40/100 and right from 70/100 to 50/100. From a kinematic and electromyographic point of view, the parameters that had major variations between before and after the pulsed radiofrequency procedure are reported below. In abduction, the left ROM (range of movement) went from 100.7° to 133.4° with an increase in the percentage contribution of the anterior deltoid muscle from 22.33% to 29.66% and a reduction in the contribution of the muscle descending (or upper) trapezius from 35.28% to 23.05%. The healthy subject had a percentage contribution of 28.22% on the left and 32.86% on the right for the anterior deltoid muscle, 19.98% on the right, and 22.01% on the left for the descending trapezius muscle. There were no significant changes in right ROM in right abduction, which changed from 167 to 163°. The healthy subject presented 170° to the right and 169° to the left in abduction. In flexion, an increase in ROM from 130.3° to 179.2° on the left was obtained, with a reduction in the percentage contribution of the middle trapezius from 40.38% to 23.42% on the right. The healthy subject presented a percentage contribution of the middle trapezius of 21° to the right and 23° to the left. On the right side, the ROM went from 151° to 154°. The healthy subject showed 180° to the right and 179° to the left. In rotation, there was an increase in ROM on both the right and left sides, which went from 122.6° to 132.5° and from 106° to 122.9°, respectively. The healthy subject presented 135° to the right and 138° to the left. The percentage contribution in the rotations of the left posterior deltoid muscle went from 24.12 to 40.19% and that of the latissimus dorsi muscle from 7.62 to 15.57%.

The healthy subject had a percentage contribution of the posterior deltoid muscle of 39.72% on the right and 42.58% on the left and of the latissimus dorsi of 13% on the right and 15% on the left. The results described above in terms of percentage contribution were also confirmed when expressed in terms of% of MVIC and not only as absolute values. As regards the coactivations, an increase in the Rudolph value greater than 50% was found in the following muscle pairs: upper and lower trapezius in forward left abduction (from 14.26 to 26.67 – healthy subject 24.72), infraspinatus and teres major in abduction to the right (from 4.72 to 11.93 forward and from 5.65 to 12.18 back; healthy subject 18.18 forward and 12.55 back) and to the left (from 14.31 to 30.89 outward, healthy subject 14.95), in flexion to the right infraspinatus and teres major (from 5.55 to 23.92 outward and from 5.05 to 8.96 in return; healthy subject 15.79 outward and 8.8 in return) and on the left (from 14.6 to 23.89 in outward and from 8.96 to 12.36 in return; healthy subject 11.1 in outward and 5.63 in return); in the rotations between teres major and right infraspinatus (13.41 to 24.84 forward, healthy subject 13.39); and between latissimus dorsi and clavicular head of pectoralis major on the left (from 1.49 to 6.95 going and from 1.74 to 8.04 returning; healthy subject 0.66 going and returning). Reductions of more than 50% in coactivations were also observed, in particular in abduction between pectoralis major and right middle trapezius (from 3.19 to 1.84 forward, healthy subject 0.58 forward and 0.43 return) and on the left (from 12.24 to 3.84 going and from 8.51 to 2.96 returning healthy subject 0.91 and 0.77). In flexion, there was a decrease of more than 50% in the coactivations between the left upper and lower trapezius (from 33.97 to 21.09 forward, healthy subject 15.14), between the infraspinatus and left teres major (from 12.14 to 4.45 outward and from 8.28 to 3.39 in return, healthy subject 11.1 and 5.63), and in the rotations between the posterior deltoid and the middle trapezius to the left (from 19.62 to 8.67 forward and from 15.23 to 6.88 backward; healthy subject 11.23 and 13.86). In all these cases the dynamic analysis in graphical form made it possible to identify the exact moments, in the phase of the outward and return cycle, in which the differences in coactivation manifested themselves most. A follow-up visit from the patient after a further 6 weeks confirmed the clinical results achieved in terms of NRS, SPADI, and ROM.

Main sEMG data are also reported in Tables 1–4.

**TABLE 1:** NRS, SPADI index results before and after pulsed radiofrequency.

Side	NRS pre (R/M)	NRS post (R/M)	SPADI pre	SPADI post
Right	8/8	4/5	70/100	50/100
Left	8/8	3/4	80/100	40/100

**TABLE 2:** Patient sEMG data before (pre) and after (post) pulsed radiofrequency treatment: abduction movement.

Abduction % contribution	Right pre	Right post	Left pre	Left post	Healthy Right	Healthy Left
ROM (range of movement°)	167 ± 2,5	163 ± 3,2	100,7 ± 5,6	133,4 ± 1,6	170	169
Anterior deltoid	33,16	31,73	22,33	29,66	32,86	28,22
Middle deltoid	39,03	30,95	38,53	30,77	27,05	29,27
Upper trapezius	13,63	20,64	35,28	23,05	19,98	22,01
Lower trapezius	14,18	16,68	3,95	16,51	20,11	20,5
Pectoralis major	5,74	6,6	8,01	5,91	3,89	4,13
Middle trapezius	49,61	43,9	29,06	30,24	40,78	43,21
Infraspinatus	28,16	33,24	39,46	34,25	26,24	23,59
Teres major	16,48	16,26	23,47	29,6	29,09	28.07

**TABLE 3:** Patient sEMG data before (pre) and after (post) pulsed radiofrequency treatment: flexion movement.

Flexion % contribution	Right pre	Right post	Left pre	Left post	Healthy Right	Healthy Left
ROM (range of movement°)	151,4 ± 2,6	154,7 ± 1,8	130,3 ± 3,9	179,2 ± 2,5	180	179
Anterior deltoid	46,73	36,96	40,06	31,02	33,66	33,91
Middle deltoid	29,43	16,29	25,27	24,68	26,55	27,18
Upper trapezius	10,17	18,51	27,53	30,62	24,91	25,15
Lower trapezius	13,67	18,22	7,14	13,68	14,86	13,76
Pectoralis major	8,27	10,36	10,61	18,04	6,99	9,63
Middle trapezius	40,38	23,42	23,86	16,02	21	23
Infraspinatus	30,63	40,79	32,87	33,96	31,04	31,15
Teres major	20,72	25,41	32,66	37,98	40,89	36.22

**TABLE 4:** Patient sEMG data before (pre) and after (post) pulsed radiofrequency treatment: humeral rotation.

Rotation % contribution	Right pre	Right post	Left pre	Left post	Healthy Right	Healthy Left
ROM (range of movement°)	122,6 ± 2,9	132,5 ± 3,2	106,3 ± 6,1	122,9 ± 1,6	135	138
Teres major	18,17	13,87	23,77	18,69	29,25	16,38
Infraspinatus	33,82	34,78	37,1	25,94	19,49	25,37
Posterior deltoid	25,62	25,89	24,12	40,19	39,72	42,58
Middle trapezius	22,39	25,46	15,01	15,18	11,55	15,67
Pectoralis major	14,34	15,73	12,64	11,9	8,33	9,1
Teres major	59,4	61,79	69,55	61,58	67,56	63,9
Pectoralis major clavicular head	12,09	11,02	10,14	10,95	11,1	12,1
Latissimus dorsi	14,18	11,46	7,62	15,57	13	15

## Discussion

In the analyzed case, it was decided to integrate a kinematic and electromyographic analysis protocol already studied in previous experiences on shoulder pathology (Bongiorno et al., 2023a) with the indexing of muscle activity on MVIC and with the study of muscle coactivations, which can potentially offer physiotherapists important clinical support (Brookham and Dickerson, 2014, 2016). The decision to resort to pulsed radiofrequency from an analgesic point of view was due to the poor tolerability of the patient described later both to drugs for neuropathic pain (serotonin reuptake inhibitors) and somatic sensory pain (opiates). Acquisitions after the pulsed radiofrequency procedures indicated important changes in the motion kinematics and surface electromyography results of the subject patient. Since this is a new analysis protocol and a new approach to cancer pain treatment, the data were compared with those of a healthy subject, as a preliminary analysis for subsequent randomized controlled trials. The results indicate that, after treatment with pulsed radiofrequency on the left side, the site of previous breast surgery, the patient recovered in terms of ROM compared to the healthy subject, with a modification of some parameters relating to the muscle percentage contribution which also tend to approach the healthy subject of reference.

The range of movement (ROM) improved, still on the left side, by 33% in abduction, 38% in forward flexion, and 16% in rotation. Regarding the percentage of muscular contribution compared to the healthy subject in abduction, the various contributions approached those taken as reference after the procedure, except in the case of the middle trapezius, which continued to be activated to a lesser extent in the patient. In forward flexion, no significant variations were observed in the middle deltoid, upper trapezius, and infraspinatus (with the patient’s parameters continuing to be similar to the healthy subject); an improvement was noted in the other muscles except for the major pectoral, which was activated much more, and the middle trapezius, which was activated to a lesser extent than in the control. In rotations, all muscular contributions approached the reference subject after treatment or were already similar before, except for the major pectoral, which was activated more. This leads us to hypothesize a personalized physiotherapeutic path focused mainly on the middle trapezius and major pectoral muscles as the target of intervention. The importance of working on the pectoralis major muscle can also be inferred from the lower percentage improvement in the range of motion (ROM) achieved by the analgesic treatment in the rotational movement compared to flexion and abduction. This is because it involves this muscle to a significant extent. Comparing our results with the reference paper from 2020 (Prieto-Gómez et al., 2020), we observe that in the abduction movement, the middle deltoid muscle and the upper trapezius muscle tend to be more activated in patients with breast neoplasms, with or without pain, compared to healthy subjects, while the lower trapezius muscle is activated less than the control. This result is confirmed by our findings, both in absolute terms and in trends before and after treatment with pulsed radiofrequency. In the flexion movement, the anterior deltoid muscle and the upper trapezius muscle tend to be more activated in patients with breast neoplasms, with or without pain, compared to healthy subjects, while the lower trapezius muscle is activated less than the control. This result is confirmed by our findings, both in absolute terms and in trends before and after treatment with pulsed radiofrequency. In the rotation movement, the posterior deltoid muscle tends to be less activated in patients with breast neoplasms, with or without pain, compared to healthy subjects. This result is confirmed by our findings, both in absolute terms and in trends before and after treatment with pulsed radiofrequency. However, it is worth noting that the mentioned research group did not analyze the pectoralis major muscle in their article.

**FIGURE 4:**
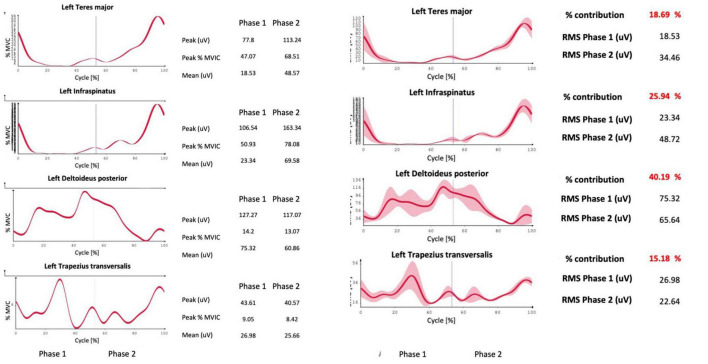
Example of a report generated by the system, including the percentage contribution of the investigated muscles to the movement and their activation in percentage ratio to the MVIC.

The analysis of the coactivations, on the other hand, describes a different story, in which the patient presents some decidedly different coactivation indices compared to those of the healthy reference subject. Analyzing the coactivations, the most interesting data pertained to rotations. Indeed, we observed an increase in coactivation between the latissimus dorsi and pectoralis major muscles after radiofrequency treatment compared to the healthy control, and a decrease in coactivation between the posterior deltoid and middle trapezius muscles. A possible explanation for the first phenomenon could be attributed to the reduced activity of the pectoralis major after analgesic treatment, with values closer to those of the healthy volunteer. This might allow the muscle to work more effectively in synergy with the latissimus dorsi (not affected by analgesic treatment and electromyographically similar, before and after the procedure, to the control subject). As for the other muscle pair, the significant increase in activity of the posterior deltoid after analgesic treatment might compensate for starting from very low values, requiring more time to develop effective synergy with the middle trapezius, despite the good electromyographic values in comparison to the control subject. This could explain the decrease in their coactivation index. It is challenging to compare results with the literature as few studies have focused on this aspect of breast cancer. The main one (Brookham and Dickerson, 2016) investigated rotations in women who had undergone breast surgery, comparing them with healthy subjects and evaluating shoulder muscle coactivations. The authors reported greater activation of the pectoralis major muscle in women with breast cancer compared to others, citing possible damage due to surgery and radiotherapy in this patient group, a result consistent with the findings of the present study. The analysis offered by the trend of this phenomenon of co-contraction over time can allow the physiotherapist to act with extreme precision with manual therapy and with therapeutic exercise on the reconstruction of the lost movement (Assila et al., 2020; Leonardis et al., 2021). Based only on the ROM and muscle percentage contribution indices, which can also be deduced with other methods [for example, with simple clinical instruments such as an inclinometer (Dougherty et al., 2015)], the physiotherapist could instead limit himself to routine treatments, deceived by the trends of these parameters. These data, in addition to being a further confirmation of the usefulness of pulsed radiofrequency treatment to allow the rehabilitation process of patients suffering from shoulder pathology after breast surgery, can be of help to the physiotherapist. The potential of the proposed protocol is mainly due to its engineering. Firstly, these analyses do not require the use of a synchronized camera to identify the start and stop moments of the cyclicity in the movement, analyzing the data acquired faster, on the one hand, and on the other partially automated since the events are determined by the accelerometer, as in other previous experiences of the authors in the sports field in the field of speed skating (Bongiorno et al., 2022b,2023b; Bongiorno and Miceli, 2023). In fact, the system already automatically identifies the moments of cycle start, movement phase inversion, and maximum movement peak, starting from the triaxial inertial sensor positioned at the mid-humeral level. The operator can then accept or modify these moments from the PC. From this, the system builds the kinematics of the movement, measuring the average value on the analyzed cycles of the ROM (range of movement) of the movement studied and the Jerk index to evaluate the fluidity of the movement itself. As far as the electromyographic component is concerned, the system requires first to acquire the MVICs of the groups of four muscles investigated, through a specific movement of flexion of the humerus, abduction, or rotation of the same Figure 4. The system also requires that all four probes are connected to the patient during MVIC tests and can read all four signals simultaneously in search of the maximum peak, should it fall not in the movement considered most specific for that muscle but during isometric contraction specific to another muscle. Here too the operator can accept the values proposed by the system or modify the maximum peak moment when generating the report. From here on, the analysis proceeds automatically, generating the final report in about 2 min. A third strong point of the system is the visualization of the coactivations graphically over time, to understand not only how much two muscles act in synergy or antagonism, but also when (Ranavolo et al., 2015). Furthermore, the fact of breaking down the three movements of the shoulder into three separate reports (for flexion, abduction, and rotation of the humerus) allows one or two of them to be used, if necessary, without necessarily having to perform them all. Finally, the system allows for a portable laboratory, since it does not require fixed cameras or power supply, and this allows it to be potentially bedside or usable in telemedicine as a periodic monitoring tool for telerehabilitation in the oncological field, which is already a reality at the national cancer center of Aviano (Biancuzzi et al., 2022; Miceli et al., 2022). The study also has limitations, described below. The study was limited to a single oncological patient and aims to be the presentation of a path that brings research closer to the clinic with a relatively simple tool to use and a cultural stimulus rather than a real therapeutic aid in this initial phase. A greater sample size will be needed in a forthcoming randomized controlled trial to obtain robust data that can guide physiotherapy pathways in breast cancer disease. A further limitation is the fact that the follow-up period is limited to about 12 weeks, while pulsed radiofrequency can give longer-lasting results, even after several months, and that, consistent with what is stated in the introduction, fibromyalgia is not one of the pathologies that responds most to this type of treatment. Furthermore, the data object of the kinematic protocol requires about an hour to be collected and processed integrally, even with the proposed semi-automated algorithm, so the organization of the department must take these times into account. In conclusion, as part of the search for a personalized rehabilitation program underway at the national cancer center in Aviano, a new kinematic and electromyographic analysis protocol of the shoulder was developed and tested for patients operated on at the breast (Bongiorno et al., 2022a). Starting from previous experiences with patients and athletes, the algorithm in use has been modified by adding the indexing of the electromyographic values on MVIC, the description of the coactivations statically and dynamically, and the greatest simplification and speed possible in the use, potentially even at the bedside, to make the reports produced comparable with those of other subjects or the same subject at different times. Pulsed radiofrequency has been indicated as a potential simple, safe, and effective pain control technique, to accompany patients, with a single treatment, for several weeks in their rehabilitation process. The visualization of the coactivations, especially in a graphic way, can be an important tool to support the physiotherapist where ROM and muscle percentage contribution may not be enough. In this co-production path in which the authors were protagonists (Biancuzzi et al., 2019, 2020; Cobianchi et al., 2022) the physiotherapist, the orthopedist, the pain doctor, the patients, and the athletes contributed to generating not only an analysis protocol but a new working method working together, each offering their skills and knowledge. This method does not want to be an absolute dogma in the results presented, which will be verified soon, but “a stone’s throw in the pond” of movement analysis, to stimulate and support the researchers who dedicate themselves to it every day. In the future, patients may benefit from a non-invasive, simple analysis tool able to lead to a personalized rehabilitation plan based on their specific residual impairment after cancer treatments. The physiotherapist will potentially be able to work where there are more pronounced joint or muscle problems, rather than relying solely on generic treatment protocols. Finally, to disseminate this type of knowledge, it is necessary to create a greater culture of movement analysis in the world of physiatrists and physiotherapists, so that centers capable of performing such complex analyses can also be useful for patients from other geographical areas where they are not performed, to personalize oncological rehabilitation that goes beyond the proximity service and uses a common language between the various health professionals involved in the process.

## Data availability statement

The raw data supporting the conclusions of this article will be made available by the authors, without undue reservation.

## Ethics statement

Ethical approval was not required for the studies involving humans because not required in our country for single case reports. The studies were conducted in accordance with the local legislation and institutional requirements. The participants provided their written informed consent to participate in this study. Written informed consent was obtained from the individual(s) for the publication of any potentially identifiable images or data included in this article.

## Author contributions

GB: Conceptualization, Investigation, Writing−original draft. AT: Supervision, Validation, Writing−review and editing. GV: Formal Analysis, Supervision, Writing−review and editing. AR: Validation, Visualization, Writing−review and editing. HB: Formal Analysis, Supervision, Writing−review and editing. FD: Methodology, Supervision, Writing−review and editing. RB: Supervision, Validation, Visualization, Writing−review and editing. LM: Conceptualization, Writing−original draft.
